# Fiber Bridging Induced Toughening Effects on the Delamination Behavior of Composite Stiffened Panels under Bending Loading: A Numerical/Experimental Study

**DOI:** 10.3390/ma12152407

**Published:** 2019-07-28

**Authors:** Angela Russo, Mauro Zarrelli, Andrea Sellitto, Aniello Riccio

**Affiliations:** 1 Department of Engineering, University of Campania Luigi Vanvitelli, via Roma, 29, 81031 Aversa, Italy; 2 Institute of Polymers, Composites and Biomaterials, CNR—Research National Council of Italy, P.le E. Fermi, Granatello, Portici, 80055 Naples, Italy

**Keywords:** fracture toughness, delamination, ultrasonic inspection, numerical simulations, carbon fibers

## Abstract

In this paper, a research activity, focused on the investigation of new reinforcements able to improve the toughness of composite materials systems, is introduced. The overall aim is to delay the delamination propagation and, consequently, to increase the carrying load capability of composite structures by exploiting the fiber bridging effects. Indeed, the influence of fiber bridging related Mode I fracture toughness (*G_Ic_*) values on the onset and propagation of delaminations in stiffened composite panels, under three-point bending loading conditions, have been experimentally and numerically studied. The investigated stiffened panels have been manufactured by using epoxy resin/carbon fibers material systems, characterized by different *G_Ic_* values, which can be associated with the material fiber bridging sensitivity. Experimental data, in terms of load and delaminated area as a function of the out-of-plane displacements, have been obtained for each tested sample. Non-Destructive Inspection (NDI) has been performed to identify the debonding extension and position. To completely understand the evolution of the delamination and its dependence on the material characteristics, experiments have been numerically simulated using a newly developed robust numerical procedure for the delamination growth simulation, able to take into account the influence of the fracture toughness changes, associated with the materials’ fiber bridging sensitivity. The combined use of numerical results and experimental data has allowed introducing interesting considerations of the capability of the fiber bridging to substantially slow down the evolution of the debonding between skin and reinforcements in composite stiffened panels.

## 1. Introduction

When composite materials are applied to aerospace, one of the main, safety-driven, design objectives is to increase the capability of the structures to withstand the operating loads when accidental damages occur. The improvement of this capability, actually, can be achieved by adopting a Damage Tolerant Design approach [1].

Indeed, laminated structures often are characterized by inter-laminar failures (delaminations and debonding), arising from pre-existing manufacturing defects, stress concentration, impact with foreign objects, and which can rapidly evolve towards structural collapse due to global or local buckling phenomena [2,3,4,5,6,7]. A suitable study of the delamination phenomenon must take into account all the parameters governing the delamination behavior, including the toughening mechanisms, such as the fiber bridging, which can play an important role in the delamination growth. According to the fiber bridging phenomenon, the fibers cross the crack faces and slow down the delamination growth. Mode I fracture toughness (*G_Ic_*) trend as a function of the crack length, also known as resistance curve (R-curves), can give a measure of the fiber bridging phenomenon. Indeed, an increase of Critical Energy Release Rate with crack length is representative of the effects of the bridging in terms of toughening.

Therefore, as for the fiber-reinforced concrete [8,9,10], taking advantages in design from the fiber bridging phenomenon as tailored through-the-thickness reinforcement could represent a new frontier for research on damage tolerance of carbon fiber reinforced epoxy resin and could lead to the significative reduction of classical metal joints [11,12,13]. The fiber bridging phenomenon and the correlated R-curve behavior have been extensively studied in the literature. Bao et al. in [14] demonstrate that the fibers interactions with the crack edges have the desirable effect of suppressing delamination. In [15], an experimental campaign to study the role of the fiber bridging in the delamination evolution retardation in composite laminates is presented. The R-curves behavior, due to the bridging, has been experimentally investigated by Sørensen and Jacobsen in [16] for unidirectional carbon fiber/epoxy resin composite specimens. In [16], the effects of fiber bridging are shown to be dependent on the specimen geometry. Such dependence has been also assessed in [17] for unidirectional composite Double Cantilever Beam (DCB) specimens. Indeed, the experimental results confirm that, even if the initial energy release rate value can be considered as a material characteristic, the shape of the R-curve strongly depends on the geometrical parameters. The influence of the fibers’ orientation at the delamination interface on the Mode I fracture toughness of multidirectional composite laminates, with special stacking sequences, able to maintain the whole elastic behavior of the specimens, has been studied in [18]. Large scale bridging phenomenon has been found in the specimens with 45°//45° crack interface, concerning the 0°//0° interface. Such results have been confirmed in [19], where the plateau value of the *G_Ic_* for the 45°//45° plies interface has been found 70% higher than the one found for 0°//0° plies interface. As a matter of fact, in literature, the dependence of the fiber bridging phenomenon on geometric parameters and stacking sequence of the laminate have been extensively studied. However, limited works are available on the relation between the fiber bridging phenomenon related and the inherent resin properties and composite manufacturing process. 

In the present manuscript, epoxy resin/carbon fibers coupons, manufactured using different curing processes, able to emphasize the fiber bridging sensitivity, have been inspected under flexural loading conditions. The analyzed samples are characterized by the same elastic mechanical properties but different resistance curve (R-curve) behavior, which changes according to the fiber bridging sensitivity. Each coupon is made of a skin panel bonded to a stringer foot. Experimental three-point bending tests [20,21,22] have been performed to assess the fiber bridging effects on the separation between the skin and the reinforcement. After the mechanical test, the damaged specimens have been inspected by adopting an ultrasonic Non-Destructive Technique (NDT) [23,24] to measure the delaminated area and assess the depth of the damages. Finally, the experimental tests have been numerically simulated using a robust finite elements procedure able to mimic the delaminations propagation by taking into account the changes in critical energy release rate with the crack length and, hence, the fiber bridging phenomenon [25]. The experimental and numerical results have been found very useful to improve the understanding of the effect of the materials’ fiber bridging sensitivity on the skin-stringer separation phenomenon in stiffened composite panels.

In Section 2, the material model, adopted to numerically simulate the fiber bridging phenomenon, is described; while experimental and numerical procedures for the delamination growth investigation are introduced in Section 3. Finally, in Section 4, the results and comparisons are presented and discussed.

## 2. Material Model Description

**Figure 1:**
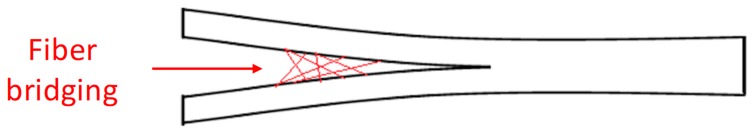
Fiber bridging schematic representation.

Epoxy resin/carbon fibers material systems, characterized by different resistance curves (R-curves), are analyzed in this paper. More in detail, the elastic mechanical properties of the investigated material systems are the same, except for the Mode I fracture toughness, which changes according to the fiber bridging sensitivity. Fiber bridging is an intrinsic phenomenon of the fiber-reinforced composite materials, which develops from delaminations, as shown in Figure 1. Many factors can influence the onset and the development of the fiber bridging, including geometric characteristics of the specimen (such as the thickness), the dimensions, and the orientation of the fibers at the delamination interface. However, also material characteristics and cure process parameters can strongly influence the fiber bridging toughening effects: actually, this is the focal point of the present paper.

**Figure 2:**
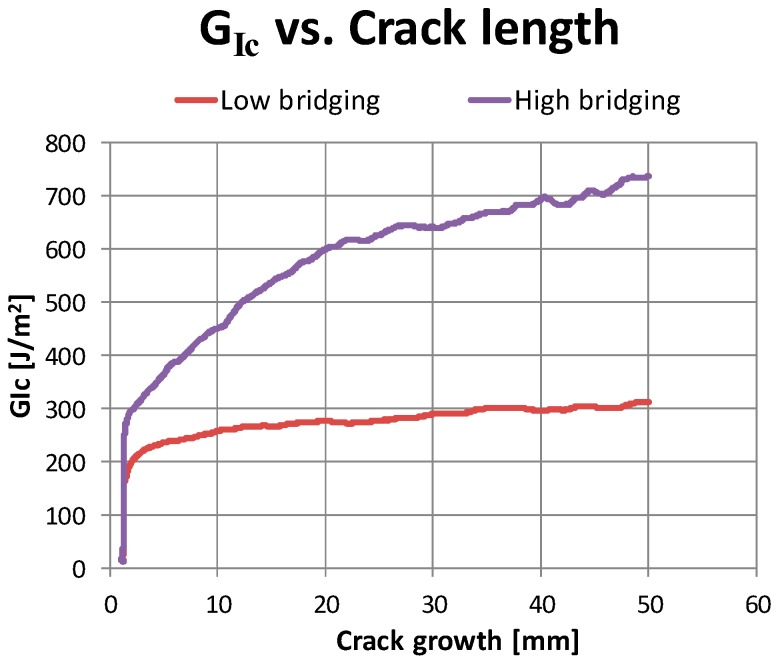
Effects of fiber bridging on *G_Ic_* vs. Crack length curves.

As literature studies have widely recognized, fiber bridging can increase the Mode I critical energy release rate, *G_Ic_*, during the crack propagation. The curve representing the crack growth resistance as a function of the crack length (Δa), named R-curve, is characterized by a “flat” trend for brittle materials when no energy-dissipating phenomena, such as fiber bridging and/or delamination migration, occur. On the other hand, in materials with “growing” R-curves, the resistance to the crack growth increases, and higher energy is needed to achieve crack propagation. This R-curve trend is representative of the fiber bridging phenomenon, which leads to a more stable delamination propagation (see Figure 2). In such materials, the *G_Ic_* value asymptotically reaches a steady-state value.

According to the chart in Figure 2, the cases of interest investigated in the frame of this paper are:

- material with low sensitivity to the fiber bridging, characterized by quasi-constants values of *G_Ic_*;
- material with high sensitivity to the fiber bridging, characterized by values of *G_Ic_* increasing with the crack length up to the asymptotic value of 0.737 kJ/m^2^.

The elastic mechanical properties of the investigated material systems are listed in Table 1. The differences in Mode I fracture toughness values are reached by using different curing processes, related to the desired sensitivity to the fiber bridging phenomenon.

## 3. Experimental and Numerical Procedure for Delamination Propagation

As already remarked, this paper is focused on the assessment of the fiber bridging induced toughening effect on skin stringer in composite stiffened panels manufactured with material systems with respectively, low and high sensitivity to the fiber bridging phenomenon. In this Section, the experimental and numerical procedures adopted for investigating the skin stringer debonding evolution in such specimen are introduced.

### 3.1. Experimental Procedure

**Figure 3:**
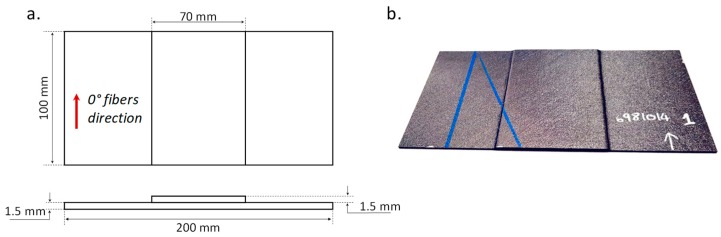
Fiber-reinforced composite coupon: (**a**) geometrical description; (**b**) picture of the tested specimen.

Three-point bending flexural tests have been performed on fiber-reinforced composite coupons, to emphasize the Mode I driven skin stringer debonding. The mechanical test procedure has been based on the ASTM D7264, which is the standard test method for flexural properties of polymer matrix composite materials [20]. The tested samples can be considered representative of a stiffened composite panel, consisting of a skin panel bonded to a foot stringer. The geometrical description of the specimens is shown in Figure 3a. As already remarked, the specimens are made of epoxy resin reinforced with carbon fibers. The stacking sequence (the same for skin and the foot stringer) is (+45, 0, −45, 90) s, with a layer thickness of 0.1875 mm and a 45°//45° skin-stringer interface. A picture representative of the tested specimen is shown in Figure 3b.

**Figure 4:**
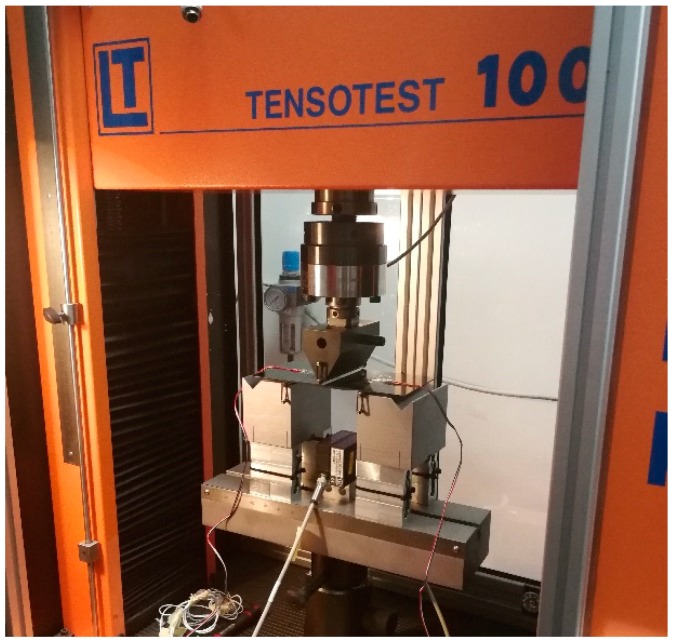
Experimental test rig.

**Figure 5:**
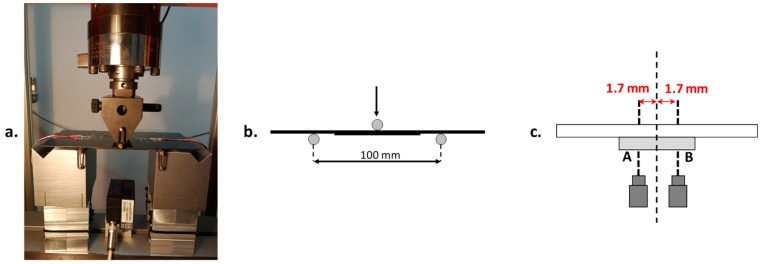
(**a**) Experimental set-up; (**b**) Test scheme; (**c**) Laser displacement sensors position.

The experimental tests have been performed using a universal testing machine Lonos Test TENSO-TEST 100 (TT100, Lonos Test Srl, Monza, Italy). The test rig is shown in Figure 4. The samples have been positioned on two lateral cylindrical supports, with a spacing span of 100 mm and with the stringer foot facing down. At the center of the skin, a controlled displacement of 1 mm/min has been applied using a cylinder actuated by a hydraulic system with a high-precision load cell of 20 kN. In Figure 5a, the experimental set-up is displayed, while in Figure 5b, the boundary conditions are schematized. Two laser displacement sensors have been used to measure the out-of-plane displacements in the stringer foot location, corresponding to point A and B in Figure 5c.

After the test execution, ultrasonic inspections have been performed with an OmniScan^®^ SX (Olympus Italia S.r.l., Segrate, Italy) ultrasonic testing device to assess the delamination extension and position. The calibration of the scanner has been carried out by considering the material parameters and the thickness of the samples, necessary to determine the velocity of the ultrasonic wave. After setting the parameters, the ultrasonic scan has been performed in water film, by moving a Phased Array (PA) probe, connected to an encoder, on the sample.

### 3.2. Numerical Procedure

**Figure 6:**
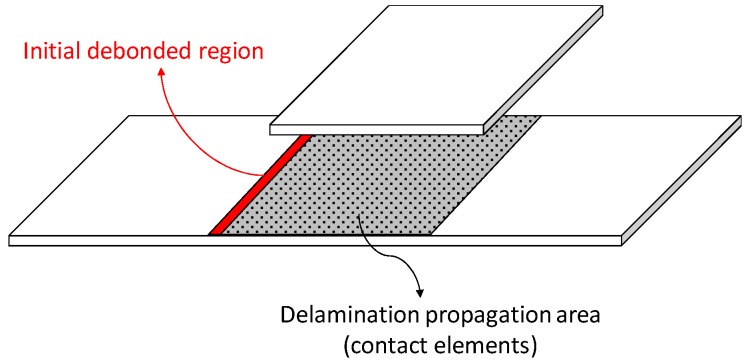
Delamination propagation area modeling.

The numerical tool adopted to perform the simulations presented in this paper, called “SMart-time XB” (SMXB), has been developed and preliminarily validated in [25]. This tool, based on a combination of the Virtual Crack Closure Technique (VCCT) and the Fail release procedure (FR), has been included in the ANSYS^®^ Finite Element Analysis (Version 2018, ANSYS, Inc., Canonsburg, PA, USA) platform by using the Ansys Parametric Design Language (APDL). The SMXB is capable of mimicking the delamination development, including the fiber bridging phenomenon by avoiding mesh and time-step dependency issues. According to the numerical procedure implemented in the SMXB tool, an initial debonded region must be considered, as for the standard VCCT-based crack simulation models. Indeed, the structure is split into two sub-laminates with a propagation region defined at their interface. The propagation area is modeled by introducing specific fracture conditions and contact elements at the interface of the two sub-laminates, as shown in Figure 6.

The Virtual Crack Closure Technique equations are used to evaluate the strain energy release rate (SERR) on the delamination front. Relations for four-noded solid elements are described in Equation (1). The growth criterion considered for the delamination propagation is the Linear Power Law (Equation (2)), which allows releasing the constraints between the pairs of nodes, in the delamination propagation area.

(1) $$G_{j}=\frac{F_{j}\Delta u_{j}}{2\Delta A}$$

(2) $$E_{d}=\frac{G_{I}}{G_{Ic}}+\frac{G_{II}}{G_{IIc}}+\frac{G_{III}}{G_{IIIc}}\geq 1$$

In Equation (1), *G_j_* (with *j* = *I, II, III*) is the energy release rate associated with the fracture mode *j*, *F_j_* is the force acting at the crack tip for the fracture mode *j*, Δ*u_j_* is the displacement at crack tip for the fracture mode *j*, and Δ*A* is the released area. In Equation (2), *E_d_* represents the failure index, *G_Ic_*, *G_IIc_*, and *G_IIIc_* are the critical energy release rates associated, respectively, with fracture mode *I*, *II,* and III, which are determined by standardized experimental tests [26,27,28].

The SMXB numerical tool simulates the delamination propagation by combining four separated and interacted moduli. The first modulus iteratively changes the size of the load step to equate the area numerically computed, *A_NUM_* in Equation (3), and the area that should be released to achieve the unit value of *E_d_*, *A_ES_* in Equation (4).

(3) $$A_{NUM}=\sum_{i=1}^{N}\Delta A_{i}^{D}$$

(4) $$A_{ES}=\sum_{i=1}^{N}\Delta A_{i}^{*}=\sum_{i=1}^{N}\left(\sum_{j=1}^{3}\frac{F_{ji}\Delta u_{ji}}{2G_{jC}}\right)$$

According to Equations (3) and (4), *N* is the number of node couples characterized by *E_d_* = 1 ⋅ $\Delta A_{i}^{D}=f\cdot \Delta A^{e}$ with *f* ≥ 1, and $\Delta A^{e}$ is defined as element size at the crack tip. The equivalence between areas (Equation (5)) is continuously checked at each load-step.

(5) $$A_{ES}≅A_{NUM}$$

**Figure 7:**
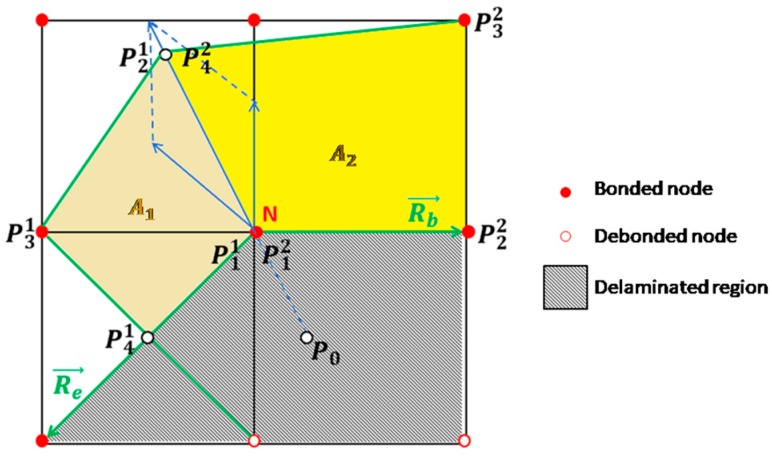
XB points and virtually closed area definition for the node N.

In the frame of the second modulus, the local coordinates systems are defined according to the instantaneous delamination front shape, to evaluate the delaminated area. Indeed, when delamination starts to grow, the delamination front shapes are modified, and the local normal direction for each node needs to be changed. According to this modulus, eight vectors (*R*_1_, …, *R*_8_) are introduced in each node of the delamination front. From (*R*_1_, …, *R*_8_), *R_e_*, and *R_b_*, boundary vectors between bonded and debonded nodes can be evaluated. Such vectors allow to define the eight points, denoted as “XB points”, in the natural coordinates system, used to calculate the virtually closed area related to each node. Figure 7 shows an example of XB points position and boundary vectors, for a certain delamination front shape.

Point *P*_0_ in Figure 7 can be calculated using the Shoelace formula, in Equation (6). Indeed, *A*_1_ + *A*_2_ is the virtually released area related to node *N*.

Since the delamination propagation can result in a non-smooth delamination front, the third modulus of the procedure has been introduced to guarantee that the growth criterion is satisfied for the node and for the segments belonging to the node, to avoid peaks in the Strain Energy Release Rate.

**Figure 8:**
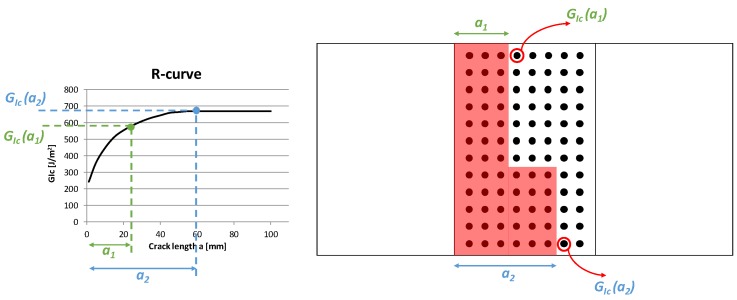
Fiber bridging modulus.

Finally, the fourth modulus, which is related to the fiber bridging evaluation, takes into account the entire R-curve (critical energy release rate as a function of the crack length) trend, with no empirical formulas or analytical approximations, to assign to each couple the nodes of the correct Mode I critical strain energy release rate value, needed for the debonding propagation, as schematized in Figure 8. According to the fourth modulus introduction, the growth criterion considered for the delamination propagation, in Equation (2), should be rewritten as a function of the crack length *a*, as described in Equation (6).

(6) $$E_{d}\left(a\right)=\frac{G_{I}}{G_{Ic}\left(a\right)}+\frac{G_{II}}{G_{IIc}}+\frac{G_{III}}{G_{IIIc}}\geq 1$$

**Figure 9:**
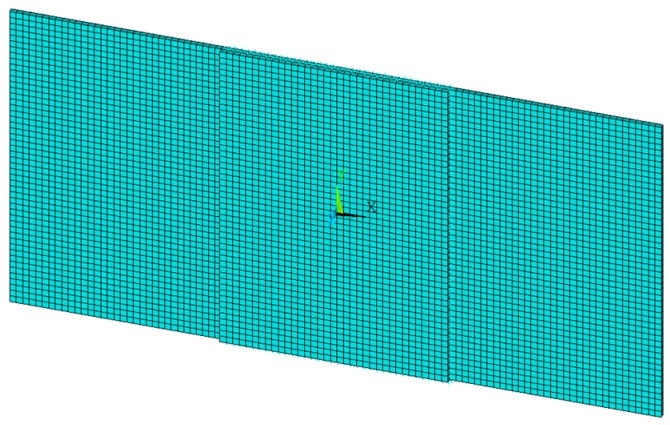
Finite element model.

The finite element model, representing the tested specimen, is shown in Figure 9. Twenty-node solid hexahedral layered elements, with 3 degrees of freedom per node, have been used. The cylindrical supports, adopted during the experimental tests, have been neglected in the FEM model and substituted by equivalent boundary conditions (suppression of the out-of-plane displacements along the lines of nodes, on the lower side of the skin, positioned at the centerline of the two cylinders). A perpendicular displacement has been applied at the mid side of the panel on the upper side of the skin to simulate the application of the bending load. 

## 4. Results, Discussion, and Comparison

In this Section, the experimental data and the numerical results are compared to assess the capability of the fiber bridging phenomenon to delay the damage evolution and to increase the maximum attained load of the tested composite specimens. For the sake of clarity, the tested specimens have been labeled as in Table 2, according to fiber bridging sensitivity.

**Figure 10:**
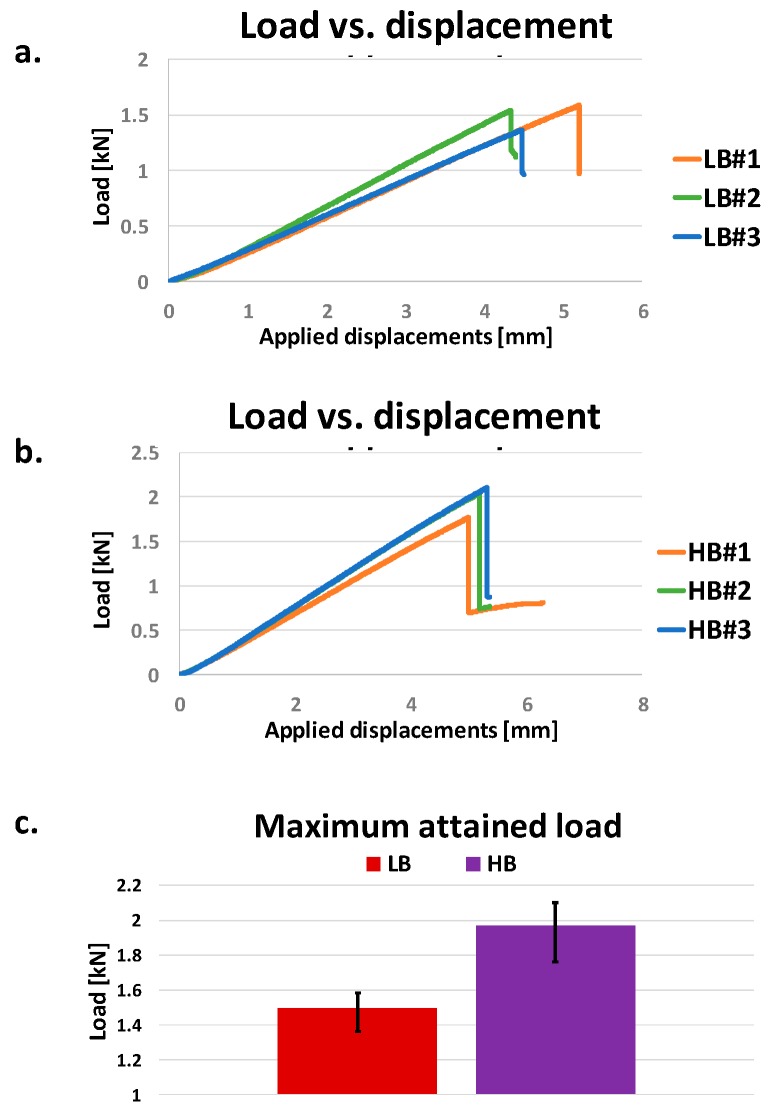
Experimental Load vs. Applied displacements in (**a**) low bridging sensitivity case, (**b**) high bridging sensitivity case; (**c**) max loads comparison. LB: low bridging; HB: high bridging.

The results of the three-point bending experimental tests, in terms of the load as a function of the applied displacement, are presented in Figure 10a,b, respectively, for the specimens with low and high sensitivity to the bridging phenomenon. Materials fiber bridging sensitivity leads to significant variations in terms of maximum load. Indeed, an increase of about 31.6% among the average values of the experimental measured ultimate loads has been found passing from low bridging sensitivity to high bridging sensitivity materials, as remarked by the chart in Figure 10c, where the data range of variation is pointed out.

**Figure 11:**
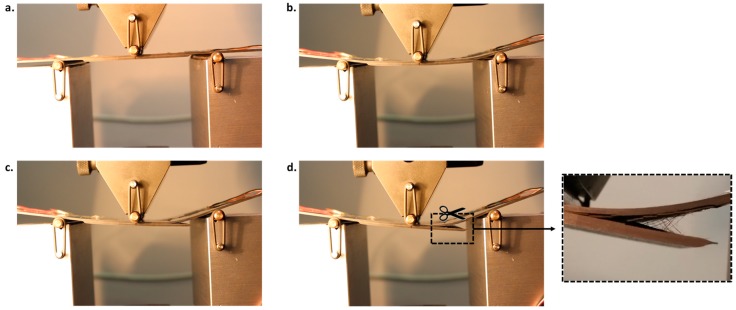
Deformed shape at: (**a**) test initiation; (**b**) debonding onset; (**c**) intermediate debonding state and (**d**) final debonding.

As an example of the flexural behavior of the analyzed panels, pictures, taken during the experimental tests on a specimen with high sensitivity to the fiber bridging phenomenon, are shown in Figure 11. A detail of fiber bridging can be observed in Figure 11d (zoom view).

**Figure 12:**
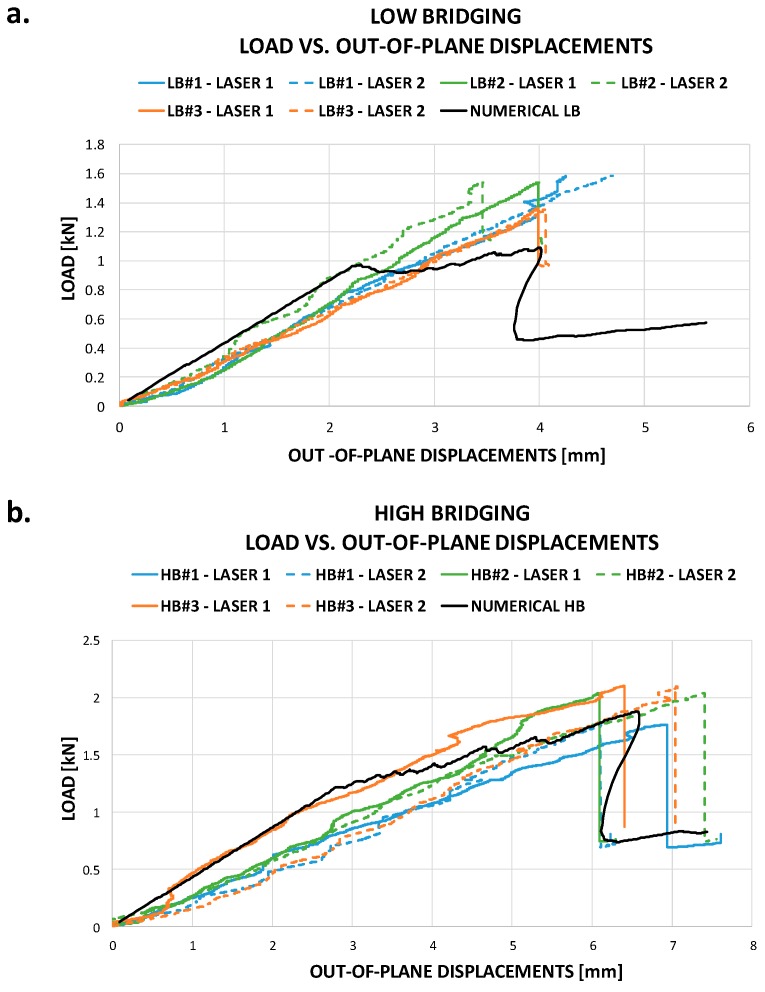
Load vs. Out-of-plane displacements comparison in (**a**) low bridging (LB) sensitivity and (**b**) high bridging (HB) sensitivity.

In Figure 12a,b, the Load vs. Out-of-plane displacements curves, experimentally obtained by laser sensors readings, have been compared to numerical results, respectively, for the material with low and high sensitivity to fiber bridging. Since the laser displacement sensors have been positioned as in Figure 5c, the discrepancy between the experimental laser 1 and laser 2 measurements, in terms of out-of-plane displacements, can be observed in Figure 12, particularly for the specimen with high sensitivity to the fiber bridging. Indeed, only one of the laser displacement sensors is located on the opening side, such as LB#1-LASER 2, LB#2-LASER 1, LB#3-LASER 2, HB#1-LASER 1, HB#2-LASER 2, HB#3-LASER 2. In such locations, the laser sensors readings could be distorted because of a specimen slip on the cylindrical supports, given by the debonding propagation. The numerical out-of-plane displacements have been extrapolated considering the position of the laser displacement sensors located on the side opposite the opening side.

For the material with low sensitivity to fiber bridging (Figure 12a), the bending stiffness well predicted by the code and the scattering between the sets of experimental data is acceptable. The maximum displacement before the complete debonding of the skin is again well predicted by the SMXB routine, but the maximum load is underestimated. The numerically predicted propagation is overestimated before the complete debonding occurs inducing an overestimated reduction of the bending stiffness if compared to the experimental data. On the other hand, the behavior of the material with high sensitivity to fiber bridging (Figure 12b) is very well predicted in terms of bending stiffness, maximum load, and maximum displacement at complete debonding of the stringer. 

Comparing the behavior of the two material systems, it is possible to notice that the increase in fiber bridging sensitivity causes an increase in the maximum load and the maximum displacement before the occurrence of the complete debonding of the stringer. Hence, the fiber bridging phenomenon can delay the skin-stringer debonding propagation, allowing to increase the capability of the panel to bear the bending load. This behavior is correctly predicted by numerical simulations. 

**Figure 13:**
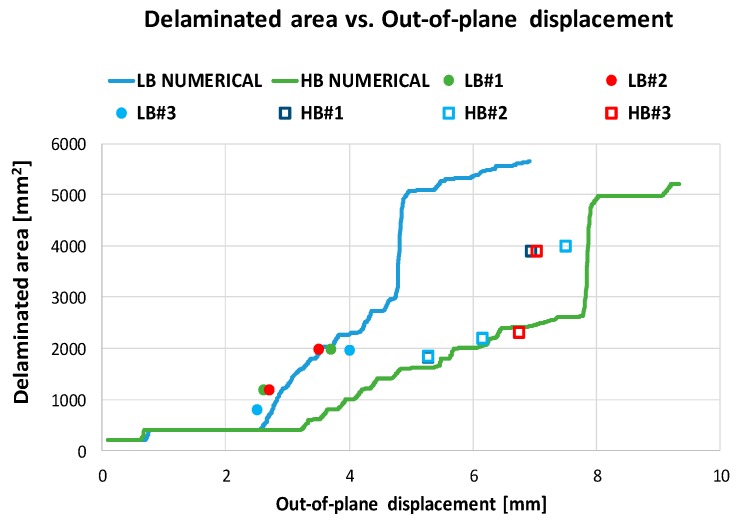
Delaminated area vs. Applied displacements, comparisons between the experimental and numerical results (low (LB) and high bridging (HB) sensitivity).

The increasing of the delaminated area between skin and stringer foot has been monitored for each analyzed configuration both experimentally and numerically during the loading process, providing an interesting overview of the material sensitivity to the fiber bridging influence on the debonding onset and stable/unstable evolution. The experimental delaminated area has been evaluated by multiplying the measured crack length by the specimen width. This measure has been considered realistic, as a first approximation, since a mostly uniform propagation along the width has been experimentally observed. Numerical results and experimental data in terms of delamination propagation as a function of the out-of-plane displacements are presented in Figure 13 for the two analyzed material configurations. According to Figure 13, the delamination propagation gets more stable as the material sensitivity to the fiber bridging increases. Indeed, the complete debonding between skin and foot stringer first occurs for the configuration with low material sensitivity to fiber bridging. The numerical predictions help us in understanding that for both the material configurations, after a first stable skin stringer debonding, a highly unstable growth takes place up to the complete debonding of the stringer foot.

**Figure 14:**
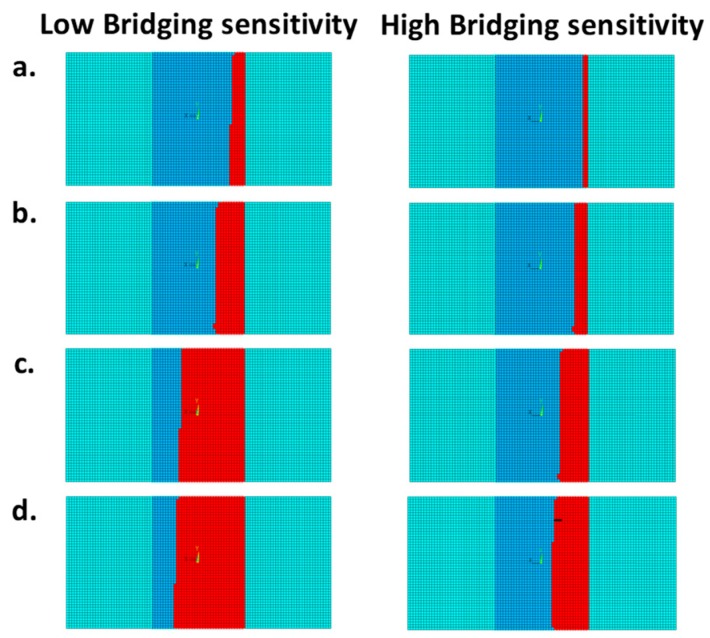
Delaminated area at: (**a**) 2.5 mm applied displacement, (**b**) 3.6 mm applied displacement, (**c**) 5.6 mm applied displacement, (**d**) 6.8 mm applied displacement. Low and high bridging sensitivity.

This behavior is confirmed by the results shown in Figure 13, in red, the numerically predicted delaminated area at different load steps for the two analyzed material configurations. The differences in delamination size evolution, between the two analyzed material systems, are highlighted in Figure 14. Indeed, considering the same applied displacement values, a decrease of the delaminated area can be observed as the material sensitivity to fiber bridging increases.

**Figure 15:**
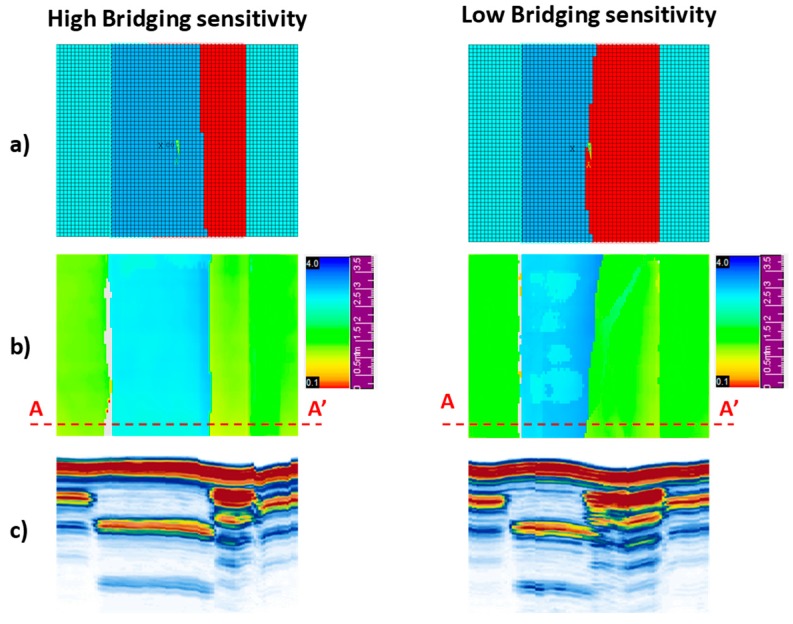
Non-destructive inspections: (**a**) numerical models, (**b**) C-scans, (**c**) S-scans.

As already mentioned, ultrasonic inspections have been performed on the tested samples. The ultrasonic scans reveal the debonding extension and the depth of the damages. Figure 15 shows the comparison between the numerically determined debonded region (Figure 15a) and the experimentally detected ultrasonic C-scan inspections (Figure 15b) for applied displacements of 4 mm, focusing on the stringer foot region: an excellent agreement between numerical results and experimental observations, in terms of delaminated area, has been found for the two analyzed material configuration. 

Figure 15c shows the S-scan inspections performed for both the material systems configurations at an applied displacement of 4 mm. The extension of the delamination along the width of the specimen (section A-A’ in Figure 15) for both the configurations are compared confirming the differences in delamination propagation observed due to the different fiber bridging sensitivity.

Based on the C-scan inspections, important consideration can be done on the debonding depth. Indeed, the rise of delaminations can be observed also in interfaces different from the skin-stringer foot interface during the loading process. This means that delamination migrates toward interfaces with different fibers orientation, characterized by a lower inter-laminar toughness.

## 5. Concluding Remarks

The effects of toughness variations, due to the fiber bridging phenomenon, on the separation between skin and reinforcement in epoxy resin/carbon fibers coupons have been, both experimentally and numerically, investigated. Material with different sensitivity to the fiber bridging phenomenon has been analyzed. Three-point bending tests have been carried out to assess the differences in terms of maximum attained load and delaminated area evolution among the different investigating material systems configurations. The experimental and numerical results have demonstrated the strong impact of the fiber bridging toughening mechanisms on the ultimate load of the panel, which increases with the fiber bridging sensitivity. Furthermore, a relevant delay in delamination evolution, when passing from materials with low to high sensitivity to the fiber bridging, has been observed. In particular, numerical simulations have allowed assessing that the increase of fiber bridging phenomenon results in more stable skin-stringer separations. Indeed, fiber bridging has been found able to lead the investigated structure to strongly increase its carrying load capability. Hence, fiber bridging appears to be an effective toughening mechanism able to delay the delamination growth phenomenon without the insertion of embedded reinforcing elements, which, usually, cause internal damages in composite microstructure and lower the stiffness and strength material characteristics.

Finally, ultrasonic inspections have confirmed the effects of fiber bridging in terms of delamination growth delay and have highlighted that delamination migrates toward interfaces with different fibers orientation, characterized by a lower inter-laminar toughness. 

## Figures and Tables

**Table 1:** Elastic mechanical properties of the investigated material systems.

Elastic Mechanical Properties	Values	Unit
E_11_	147	GPa
E_22_	8.5	GPa
G_12_ = G_13_	4.5	GPa
G_23_	4	GPa
ν_12_ = ν_13_	0.36	-
ν_23_	0.45	-
G_IIc_ = G_IIIc_	0.514	kJ/m^2^

**Table 2:** Samples denomination.

Low Bridging Sensitivity			High Bridging Sensitivity		
LB#1	LB#2	LB#3	HB#1	HB#2	HB#3
